# Contraceptive method use among women and its association with age, relationship status and duration: findings from the third British National Survey of Sexual Attitudes and Lifestyles (Natsal-3)

**DOI:** 10.1136/bmjsrh-2017-200037

**Published:** 2018-05-25

**Authors:** Nicola Firman, Melissa J Palmer, Ian M Timæus, Kaye Wellings

**Affiliations:** 1 Life Course Epidemiology and Biostatistics, Population, Policy and Practice Programme, Great Ormond Street Institute of Child Health, University College London, London, UK; 2 Centre for Sexual and Reproductive Health Research, London School of Hygiene and Tropical Medicine, London, UK; 3 Department of Population Health, London School of Hygiene and Tropical Medicine, London, UK

**Keywords:** contraception, Britain, relationship duration, age, unintended pregnancy, relationship status, national survey

## Abstract

**Background:**

One in six pregnancies in Britain are unplanned. An understanding of influences on contraceptive method choice is essential to provision compatible with users’ lifestyles. This study describes contraceptive method use by age, and relationship status and duration, among women in Britain.

**Methods:**

Data from women participating in the third British National Survey of Sexual Attitudes and Lifestyles were used to describe contraceptive use grouped as: unreliable method or none; barrier methods; oral/injectable hormonal methods; and long-acting reversible contraception. A total of 4456 women at risk of pregnancy were used to examine associations between contraception use, age, relationship type and duration. Age-stratified odds ratios for contraceptive use by relationship type and duration were estimated using binary logistic regression.

**Results:**

Some 26.0% of 16–49-year-olds used hormonal contraception as their usual method. Use of hormonal and barrier methods was highest in the youngest age group and decreased with age; the reverse was true for use of unreliable methods or none. Barrier method use was higher in short-term relationships among younger participants; this was not seen among older respondents. Duration was more strongly associated with usual contraceptive method than relationship type; this pattern was more marked among younger participants.

**Conclusions:**

Asking about relationship status and duration may help providers support women’s contraceptive use by considering their priorities and preferences at different life stages. Interactions between relationship characteristics, age and contraception are complex, and bear closer scrutiny both in research and in policy and practice.

**Key messages**

- Use of barrier methods was higher in short-term relationships among younger participants, but this was not seen among the oldest respondents.
- Relationship duration was more strongly associated with the contraceptive method used than was self-defined status of relationship, and this pattern was more obvious among younger participants.
- Asking about partnership characteristics may help providers support women in their use of contraception by considering their priorities and preferences at different life stages.

## Background

An estimated 16% of pregnancies in Britain are unplanned.^1^ Since contraception is available free of charge under National Health Service provision, and the prevalence of contraceptive use is high, efforts to reduce this number need to extend beyond addressing non-use. The risk of unplanned pregnancy resulting from method discontinuation has been shown to be almost as high as that resulting from no method use.^2^ Consequently, an understanding of influences on method choice is essential to contraceptive provision compatible with users’ lifestyles, and so less likely to be discontinued.

Studies in the United States (US) have shown both age^3 4^ and relationship characteristics to be more strongly associated with contraceptive method than other demographic factors.^5^ Contraceptive use has been associated with the number of sexual partners in Australia and Europe,^6–9^ the quality and level of trust in relationships,^5 10^ and type of relationship in Britain and the US.^11–16^ Other US studies and one in Britain have shown that condom use decreases with the relationship duration,^11–14 17 18^ while hormonal method use increases.^13–16 19^ Other studies have shown use of any method increases with relationship duration.^10 15 18^

To date, no studies using large, representative samples of women have examined how associations between method use and relationship characteristics differ by age group, or have distinguished between relationship duration and type. Further, few have taken account of fertility intentions.^13 20 21^ The aim of this study was twofold: first, to describe contraceptive method use among women in Britain in 2010–2012 by age group, and second, to examine associations in different age groups between type of partnership, duration of relationship, and contraceptive method use by women (reporting in 2010–2012) at risk of unintended pregnancy. The study uses data from the third National Survey of Sexual Attitudes and Lifestyles (Natsal-3).

## Methods

### Study design and setting

Data were analysed from Natsal-3, a cross-sectional survey of 15 162 16–74-year-old men and women living in Britain, interviewed between September 2010 and August 2012 (response rate 57.7%). The survey used a multistage, clustered and stratified probability sample. Natsal-3 data were weighted to adjust for the unequal probabilities of selection and a non-response post-stratification weight was applied.^22^

Natsal-3 was approved by the Oxfordshire Research Ethics Committee A (reference: 09/H0604/27).

### Participants

Female Natsal-3 participants of ‘reproductive age’ (aged 16 to 49 years),^23^ were included in initial analyses of contraceptive method use (and stratified by age group) for whom usual contraceptive method was reported (n=5857). To explore patterns of method use by relationship type and duration by age group, we excluded those currently pregnant, trying to conceive or menopausal (n=802), those who had not had heterosexual sex in the recent time period (n=89), and participants who relied on sterilisation (n=379) (as few such women existed in the two younger age groups). We also excluded women who did not report year of both first and most recent occasions of sex with their most recent partner (n=129), or relationship status with their most recent partner (n=2). After applying these criteria, the analysis included 4456 women (online supplementary files 1 and 2).

### Measures

#### Outcomes

**Figure 1:**
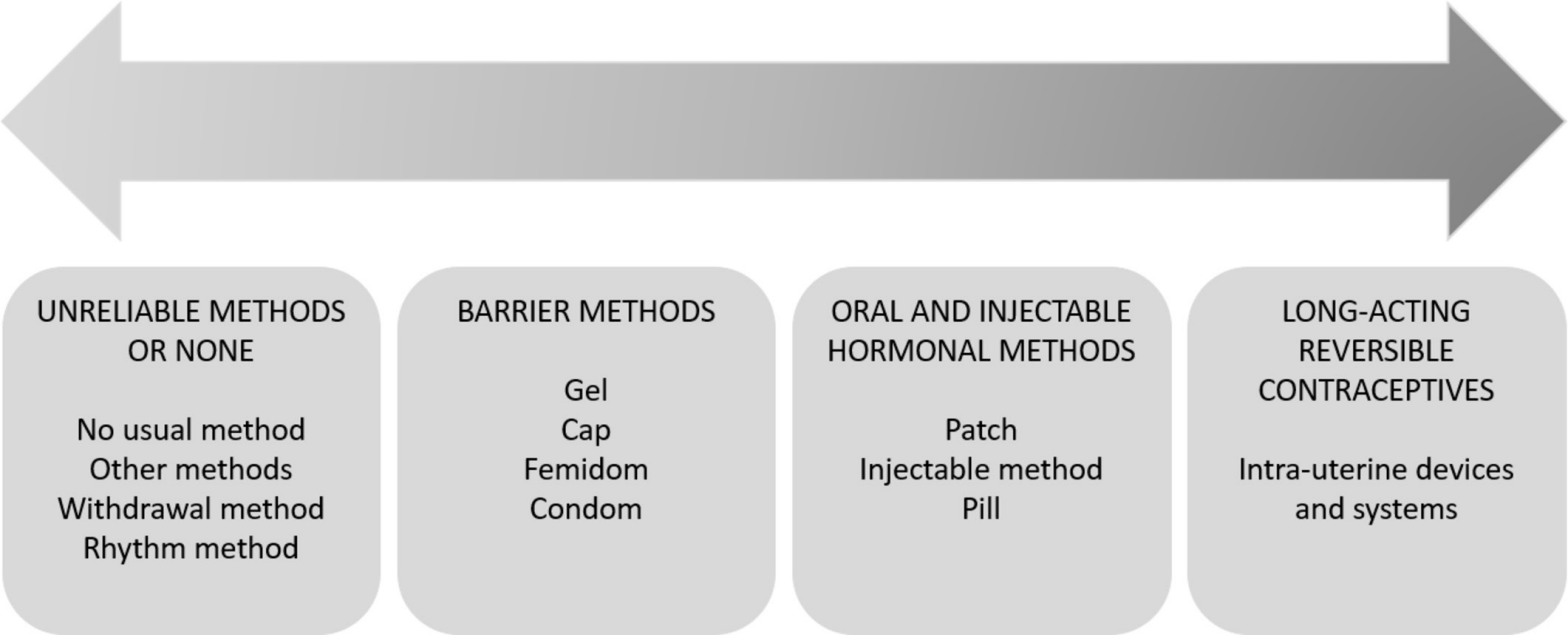
Contraceptive methods grouped by type.

The primary outcome was the contraceptive method usually used. Responses to the question ‘*Which would you say is your most usual (contraceptive) method these days?*’ were grouped into four categories (indicating type of method, as opposed to grouping based on effectiveness): unreliable or no usual method; barrier methods; oral/injectable hormonal; and long-acting reversible contraceptives (LARC) (figure 1). Participants were allowed to select up to three usual methods. For the 261 women who indicated use of more than one method, the most effective method reported was coded as their usual method.

#### Exposure variables

Relationship duration was calculated from the dates of first and most recent occasion of sex with the most recent sexual partner and grouped into mutually exclusive categories: 1 day (where the dates of first and last sex were the same); more than 1 day to 6 months; 6 months to 1 year; 1 to 3 years; 3 to 5 years; and 5 years or more.

Relationship status was derived from the question ‘*Which one of these descriptions applies best to you and [that person/name] at the time you most recently had sex?*’ Responses were grouped into four mutually exclusive categories: recently met, not steady, steady but non-cohabiting, married/cohabiting.

### Statistical analyses

Initial analyses described the association between contraceptive method group usually used and age group (16–24, 25–34 and 35–49 years), including those with limited recent heterosexual sexual experience and those currently pregnant. From this point, further analyses were restricted to women at risk of unplanned pregnancy (those *not* answering ‘I would definitely like (more) children and am currently trying’ to a fertility intention question, those pre-menopausal, and those with recent heterosexual activity). A cross-tabulation of contraceptive method type usually used and relationship status and duration showed trends in method use by relationship characteristics in different age groups.

Use of each category of contraceptive method was indicated in four dichotomous variables whereby usual use of the method of interest was coded ‘1’, else ‘0’. These dichotomous variables were the dependent variables in binary logistic regression models. Initially each binary logistic regression model predicted the crude (univariable) odds of use of the method of interest by relationship status and duration, followed by mutual adjustment for both partnership characteristic variables.

Statistical analyses were performed using Stata/SE 15 (StataCorp LP) taking into account the survey’s sample design, using the svy function.

## Results

### Contraceptive method use among all women, by age

Table 1 shows usual contraceptive method, sexual activity and pregnancy status among all women aged 16–49 years, stratified by age group. Only 6.2% had not had sex with a man in the year prior to interview, and 15.0% were either currently pregnant, trying to conceive or menopausal, totalling 21.2% unlikely to have been at risk of unintended pregnancy. The methods most commonly used by those at risk were oral/injectable hormonal methods (26.0%), barrier methods (16.4%), and unreliable methods or none (14.8%). Use of hormonal methods was highest in the youngest age group, decreasing with successive age group. The reverse was true for unreliable and no method use, which increased with age group. Reliance on sterilisation also increased with age group and was reported by almost one in five 35–49-year-olds, but by virtually no 16–24-year-olds.

**Table 1:** ‘Usual’ contraceptive method use among women aged 16–49 years, stratified by age (column percentages)

							Total		
	Age range (years) 16–24		25–34		35–49		Unweighted	Weighted	
	n	% (95% CI)	n	% (95% CI)	n	% (95% CI)	n	n	% (95% CI)
Unreliable method or none	126	7.6 (6.3 to 9.2)	295	12.3 (10.9 to 13.9)	352	19.4 (17.4 to 21.5)	773	655	14.8 (13.6 to 16.0)
Barrier methods	357	22.1 (19.9 to 24.4)	400	18.0 (16.2 to 20.0)	234	12.9 (11.4 to 14.6)	991	727	16.4 (15.3 to 17.5)
Oral/injectable methods	768	45.5 (42.7 to 48.2)	832	33.2 (31.2 to 35.2)	244	13.2 (11.5 to 15.0)	1844	1154	26.0 (24.8 to 27.3)
LARC	220	12.1 (10.4 to 13.9)	287	11.4 (10.0 to 13.0)	193	11.3 (9.8 to 13.1)	700	511	11.5 (10.5 to 12.6)
Male or female sterilisation	3	0.2 (0.1 to 0.6)	87	3.6 (2.8 to 4.5)	280	18.5 (16.4 to 20.7)	370	449	10.1 (9.1 to 11.3)
Not sexually active†	36	2.5 (1.7 to 3.5)	121	4.2 (3.5 to 5.0)	159	6.8 (5.7 to 8.1)	316	226	5.1 (4.5 to 5.8)
Only same-sex experience‡	14	1.0 (0.6 to 1.6)	24	1.0 (0.6 to 1.5)	23	1.3 (0.8 to 1.9)	61	49	1.1 (0.8 to 1.5)
Currently pregnant§ or menopausal	160	9.2 (7.8 to 10.8)	359	16.3 (14.7 to 18.1)	283	16.7 (14.8 to 18.8)	802	666	15.0 (13.9 to 16.2)
Total									
Unweighted	1684	28.8	2405	41.1	1768	30.2	5857		100
Weighted	939	21.2 (20.1 to 22.2)	1331	30.0 (28.8 to 31.3)	2167	48.8 (47.3 to 50.4)		4437	100

Proportions are weighted to account for the survey sampling design and non-response.

Total N includes women aged 16–49 years, who reported their usual method of contraception. One woman reported her usual method of contraception as an emergency method and was excluded from these results.

†Not sexually active in the last year.

‡Only same-sex sexual experience in the last year.

§Includes women currently pregnant or trying to conceive.

CI, confidence interval; LARC, long-acting reversible contraception.

### Contraceptive method use by age and relationship characteristics among women at risk of unintended pregnancy

Online supplementary file 3 gives the distribution of women by partnership characteristics. Table 2 shows contraceptive method group use by partnership characteristics by age group (online supplementary file 4 provides the crude odds ratios and online supplementary file 5 gives additional descriptive analyses of condom use by partnership characteristics).

10.1136/bmjsrh-2017-200037.supp3 Supplementary file 3

10.1136/bmjsrh-2017-200037.supp4 Supplementary file 4

10.1136/bmjsrh-2017-200037.supp5 Supplementary file 5

**Table 2:** Distribution of ‘usual’ contraceptive method use by relationship duration and relationship status, stratified by age

	Unreliable or no method	Barrier methods	Oral and injectable hormonal methods	LARC	Total			Weighted column % (95% CI)
	% (95% CI)	% (95% CI)	% (95% CI)	% (95% CI)	Unweighted n	Weighted n	Total row %	
**Age: 16–24 years**								
Relationship duration								
1 day	16.3 (11.5 to 22.5)	33.6 (27.2 to 40.5)	39.1 (32.5 to 46.2)	11.0 (7.5 to 16.0)	272	155	100	18.8 (16.6 to 21.3)
>1 day<6 months	5.2 (3.0 to 8.8)	39.2 (32.3 to 46.6)	46.2 (39.4 to 53.1)	9.4 (6.0 to 14.6)	233	126	100	15.3 (13.4 to 17.5)
≥6 months<1 year	10.6 (6.7 to 16.4)	18.9 (13.6 to 25.8)	58.4 (50.8 to 65.6)	12.1 (7.9 to 18.0)	212	117	100	14.3 (12.4 to 16.4)
≥1 year<3 years	6.4 (4.3 to 9.6)	16.4 (12.7 to 21.0)	60.0 (54.6 to 65.2)	17.1 (13.4 to 21.7)	367	204	100	24.8 (22.5 to 27.3)
≥3 years<5 years	9.2 (5.9 to 13.9)	24.0 (18.3 to 30.8)	54.5 (47.2 to 61.6)	12.3 (8.3 to 17.8)	224	127	100	15.4 (13.4 to 17.6)
≥5 years	8.1 (4.4 to 14.4)	19.3 (13.5 to 26.8)	53.0 (44.7 to 61.1)	19.6 (13.8 to 27.3)	167	93	100	11.4 (9.6 to 13.3)
Relationship status								
Recently met	13.0 (6.1 to 25.5)	44.8 (32.5 to 57.7)	31.0 (20.7 to 43.5)	11.2 (5.5 to 21.5)	70	42	100	5.1 (4.0 to 6.5)
Not steady	11.8 (8.5 to 16.3)	32.4 (27.0 to 38.2)	44.4 (38.2 to 50.8)	11.4 (8.0 to 15.9)	332	180	100	21.9 (19.5 to 24.5)
Steady*	6.8 (5.0 to 9.1)	22.2 (18.9 to 26.0)	57.7 (53.5 to 61.9)	13.3 (10.7 to 16.4)	748	398	100	48.4 (45.4 to 51.3)
Married/cohabiting	11.3 (7.7 to 16.2)	19.8 (15.5 to 25.0)	52.1 (46.3 to 57.9)	16.8 (12.8 to 21.7)	325	203	100	24.7 (22.3 to 27.2)
Total					n	n		
Unweighted (n)	133	353	771	218	1475		100	100
Weighted (n)	77 (9.3%; 7.8 to 11.1)	206 (25.0%; 22.6 to 27.6)	429 (52.1%; 49.1 to 55.0)	112 (13.6%; 11.7 to 15.8)		823	100	100
**Age: 25–34 years**								
Relationship duration								
1 day	29.9 (23.2 to 37.7)	27.5 (21.1 to 35.5)	29.1 (22.8 to 36.3)	13.2 (8.8 to 19.3)	204	105	100	10.3 (9.0 to 11.9)
>1 day<6 months	15.1 (10.1 to 22.0)	28.3 (19.3 to 39.5)	42.8 (33.9 to 52.2)	13.7 (8.8 to 20.7)	165	78	100	7.7 (6.5 to 9.2)
≥6 months<1 year	19.4 (12.0 to 29.9)	22.5 (13.8 to 34.5)	42.2 (32.6 to 52.3)	15.9 (9.9 to 24.5)	121	63	100	6.2 (5.0 to 7.6)
≥1 year<3 years	18.0 (13.0 to 24.4)	23.9 (17.3 to 32.1)	47.1 (39.2 to 55.2)	10.9 (6.9 to 16.8)	206	113	100	11.1 (9.5 to 12.9)
≥3 years<5 years	20.3 (15.4 to 26.3)	17.8 (13.2 to 23.5)	50.0 (43.1 to 57.0)	11.9 (8.2 to 17.1)	253	134	100	13.3 (11.7 to 15.0)
≥5 years	16.9 (14.4 to 19.7)	22.8 (19.8 to 26.0)	43.5 (40.2 to 46.9)	16.8 (14.2 to 19.8)	914	521	100	51.4 (48.6 to 54.1)
Relationship status								
Recently met	17.3 (10.0 to 28.3)	30.4 (19.8 to 43.6)	41.9 (30.4 to 54.4)	10.4 (5.3 to 19.3)	82	42	100	4.1 (3.2 to 5.2)
Not steady	22.7 (17.3 to 29.1)	23.6 (17.3 to 31.3)	40.7 (33.9 to 47.9)	13.0 (9.1 to 18.1)	271	128	100	12.6 (10.9 to 14.5)
Steady*	18.7 (15.0 to 23.0)	18.7 (14.7 to 23.6)	47.1 (42.1 to 52.3)	15.5 (12.0 to 19.7)	439	196	100	19.3 (17.5 to 21.3)
Married/cohabiting	18.2 (15.9 to 20.9)	24.0 (21.2 to 26.9)	42.5 (39.4 to 45.7)	15.3 (13.1 to 17.9)	1071	649	100	64.0 (61.6 to 66.3)
Total					n	n		
Unweighted (n)	357	395	825	286	1863		100	100
Weighted (n)	191 (18.8%; 17.0 to 20.9)	235 (23.2%; 20.9 to 25.6)	438 (43.2%; 40.7 to 45.7)	151 (14.9%; 13.1 to 16.8)		1014	100	100
**Age: 35–49 years**								
Relationship duration								
1 day	37.4 (26.6 to 49.5)	17.2 (9.8 to 28.5)	24.0 (15.7 to 34.7)	21.4 (13.2 to 32.9)	91	93	100	7.2 (5.7 to 9.0)
>1 day<6 months	49.5 (35.6 to 63.5)	20.4 (11.3 to 33.9)	11.8 (5.5 to 23.7)	18.3 (9.1 to 33.4)	52	42	100	3.2 (2.4 to 4.3)
≥6 months<1 year	43.5 (30.7 to 57.3)	21.0 (11.9 to 34.5)	20.3 (11.6 to 33.1)	15.1 (7.6 to 27.9)	58	45	100	3.4 (2.6 to 4.6)
≥1 year<3 years	42.7 (31.5 to 54.8)	22.4 (13.6 to 34.7)	17.9 (10.1 to 30.0)	16.9 (9.1 to 29.2)	77	67	100	5.1 (4.1 to 6.5)
≥3 years<5 years	53.8 (41.6 to 65.7)	16.4 (9.7 to 26.3)	17.7 (10.1 to 29.3)	12.1 (6.0 to 22.7)	79	86	100	6.6 (5.2 to 8.3)
≥5 years	35.7 (32.0 to 39.5)	22.3 (19.3 to 25.5)	22.8 (19.6 to 26.2)	19.3 (16.4 to 22.5)	761	974	100	74.5 (71.8 to 77.1)
	37.4 (26.6 to 49.5)	17.2 (9.8 to 28.5)	24.0 (15.7 to 34.7)	21.4 (13.2 to 32.9)	91	93	100	7.2 (5.7 to 9.0)
Relationship status								
Recently met	17.0 (7.3 to 34.9)	25.4 (11.8 to 46.3)	15.2 (6.5 to 31.7)	42.3 (24.9 to 61.9)	29	25	100	1.9 (1.3 to 2.9)
Not steady	52.5 (43.3 to 61.6)	17.3 (11.9 to 24.4)	16.3 (10.9 to 23.7)	13.9 (8.2 to 22.6)	148	125	100	9.5 (8.0 to 11.3)
Steady*	42.8 (35.4 to 50.5)	21.8 (15.7 to 29.5)	19.6 (14.1 to 26.6)	15.8 (10.9 to 22.4)	198	170	100	13.0 (11.1 to 15.2)
Married/cohabiting	36.0 (32.3 to 39.7)	21.8 (18.9 to 25.0)	23.1 (19.9 to 26.6)	19.2 (16.5 to 22.3)	743	987	100	75.5 (72.9 to 78.0)
Total					n	n		
Unweighted (n)	449	231	246	192	1118		100	100
Weighted (n)	497 (38.1%; 34.9 to 41.3)	280 (21.4%; 18.9 to 24.1)	285 (21.8%; 19.2 to 24.7)	244 (18.7%; 16.3 to 21.3)		1307	100	100

Proportions are weighted to account for the survey sampling design and non-response. Total N includes women aged 16–49 years, with at least one previous opposite- sex partner and whose most recent sexual experience was with an opposite- sex partner, who are not currently trying to conceive or are pregnant. A total of 275 women indicated more than one usual method of contraception: 232 women selected an oral and injectable method or LARC in addition to a barrier method, and the more effective method was selected as their usual method.

*Relationship status steady, but not cohabiting.

LARC, long-acting reversible contraception.

#### 16–24-year-olds

##### Unreliable and no method(s)

**Figure 2:**
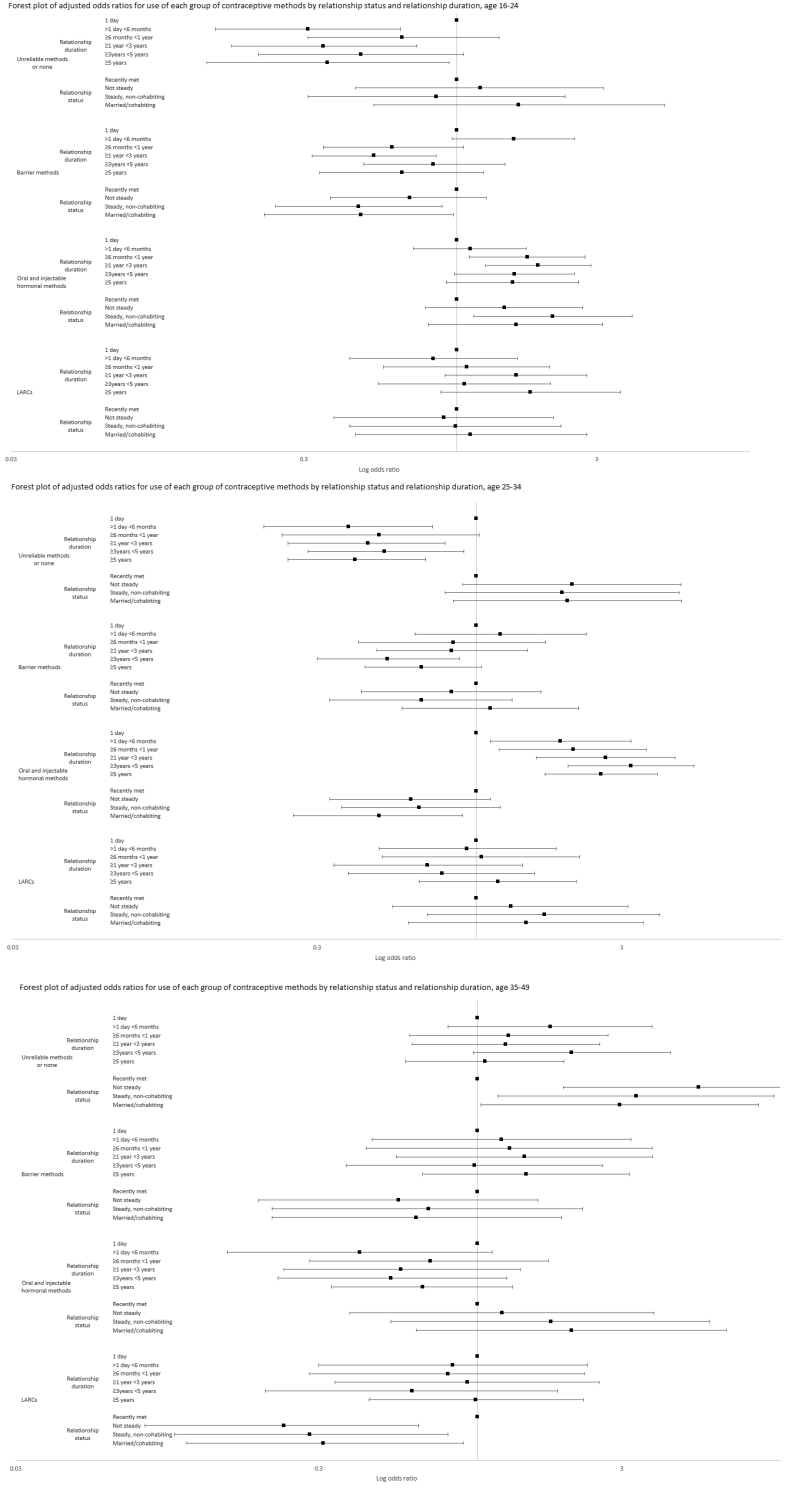
Forest plots of adjusted odds ratios for use of each group of contraceptive methods, by relationship duration and relationship status. A: 16–24 years; B: 25–34 years; C: 35–49 years.

Less than 10% of 16–24-year-olds at risk of unintended pregnancy were using an unreliable or no contraceptive method. This proportion did not vary consistently by relationship status but decreased significantly with duration. After adjustment for relationship status and duration, the association between use of an unreliable method or none and relationship duration remained significant, but the association with relationship status was weakened (figure 2A and online supplementary file 6).

10.1136/bmjsrh-2017-200037.supp6 Supplementary file 6

##### Barrier methods

Barrier method use was reported by one in four women at risk of unintended pregnancy, but decreased significantly as relationships increased in length and became more established. After adjustment, the odds of barrier method use were only significantly lower for women in relationships lasting 1 to 3 years' duration, and among women in steady or married/cohabiting relationships.

##### Oral and injectable methods

Hormonal method use accounted for over half of usual use by women at risk of unintended pregnancy. Use was highest among women in relationships of 1 to 3 years' duration and those in ‘steady’ relationships. Adjusted odds showed significant increased use of oral/injectable methods in relationships lasting between 6 months and 3 years, as well as among steady, non-cohabiting couples.

##### LARC

The proportion of women using LARC did not vary with relationship duration nor status in this age group, and this was confirmed in the adjusted logistic regression results.

#### 25–34-year-olds

##### Unreliable and no method(s)

Almost one-fifth of women in this age group used an unreliable or no method. This proportion increased with relationship duration but not status. Adjusted results showed a significant decrease in use of unreliable methods or none for all relationships lasting longer than 1 day (with the exception of relationships of 6 months to 1 year) (figure 2B).

##### Barrier methods

Almost one-quarter of women at risk of unintended pregnancy usually used a barrier method. This proportion did not vary significantly by relationship characteristics. After adjustment, only women whose most recent relationship lasted between 3 and 5 years were less likely to report use of barrier methods.

##### Oral and injectable methods

The proportion of women reporting oral/injectable method use increased with relationship duration but did not vary significantly by status. After adjustment, use of these methods significantly increased with relationship duration, but notably, decreased with relationship stability. Women whose most recent relationship had lasted only 1 day were less likely than other women to use hormonal methods, but women who said they had recently met their partner were more likely than married/cohabiting women to have used such methods.

##### LARC

There were no significant associations between use of LARC and relationship duration or status.

#### 35–49-year-olds

##### Unreliable and no method(s)

Some 38.1% of women in this age group reported usual use of an unreliable or no method. This proportion varied by relationship status but not duration. Adjusted odds ratios showed women who had recently met their partner were less likely to use an unreliable or no method (figure 2C).

##### Barrier methods

Use of barrier methods varied with neither relationship status nor duration.

##### Oral and injectable methods

The adjusted odds of using an oral or injectable method were not significantly associated with relationship status nor duration.

##### LARC

Use of LARC varied significantly by relationship status, but not duration. After adjustment, women who had recently met their partner were significantly more likely to use LARC than women in other types of relationship.

## Discussion

### Principal findings

These data from a nationally-representative survey of sexually-active women in Britain illustrate differences in method use by the nature and duration of the relationship with the most recent sexual partner and how the extent of these differences varies with age. Use of barrier methods was appreciably higher in short-term than longer-term relationships among younger participants, but this pattern was not seen among respondents in the oldest age group. While there was considerable correspondence between the duration and type of relationships and their association with contraceptive method use, some differences were observed. Relationship duration was marginally more strongly associated with the contraceptive method used than was relationship type, and this pattern was more obvious among younger participants. Moreover, relationship type and duration operated differentially in their influence on hormonal method use among 25–34-year-old women.

Comparison of our findings to those of other studies is difficult because of differences in methodology, in the definition of relationship types and in the categorisation of contraceptive methods. Typically, studies that have compared contraceptive method use in different age groups,^4 24^ have not taken relationship characteristics into account and, conversely, studies that compared contraceptive method use by relationship characteristics have not done so in different age groups.^6–10 13 14 16 25^ These differences in study design are likely to partially explain the equivocal nature of findings of many previous studies^14^ and also apparent anomalies between other studies and our own. At the aggregate level, we did not find higher levels of LARC use among younger participants than older ones, as others have.^26 27^ Nevertheless, their use increased markedly among 16–24-year-old women whose relationship had lasted 5 or more years; equivalent to the prevalence among 35–49-year-olds.

### Study strengths and weaknesses

Our large population-based dataset enabled us to estimate the prevalence of contraceptive use in different age groups by relationship type and duration, and to exclude women not apparently at risk of unplanned pregnancy. The study also has limitations. For multi-partnered participants, cross-sectional data are unsuited to tracing pathways of use and consequently we have made several assumptions in this article. First, we have assumed that respondents used their ‘usual’ method of contraception with their most recent sexual partner. When respondents reported several usual methods, the most effective may not have been the method used for the most recent sex. Second, the most recent sexual partner may not have been the main sexual partner during the previous year. Relationship duration was calculated objectively as the time between first and most recent sex with the respondent’s most recent sexual partner. This measure might not truly reflect relationship duration as it would be defined by the participant. We restricted our analyses to women, as misreporting of usual contraceptive method may be more common by men, since the majority of the methods are operationalised by women. We were limited to use of data generated by the questions asked in Natsal-3, which did not probe affective attributes of the relationship which others have linked to contraceptive method use.^5 10^ Finally, while we have no reason to believe contraceptive practices might have changed in the time since interview, these analyses are based on data reported by participants up to 8 years ago.

### Implications for policy and practice

Our data should be interpreted in the context of women’s lives, and their evolving priorities and preoccupations through the life course. Younger women, and those in more transient relationships, may be more motivated to avoid pregnancy than those who are older and in more stable relationships. Thus, the fact that one in six 16–24-year-olds uses an unreliable/no method of contraception with a partner they have just met is of concern. So, also, is the relatively low prevalence of reliable method use in shorter-term relationships among older participants. Alternatively, it is likely that although not explicitly trying to conceive, for some women a pregnancy would not be unwelcome, resulting in some ambivalence in relation to contraceptive use.^28^ An acknowledgement of pregnancy intention as a continuum is important to further understand women’s contraceptive needs. To date, sexual health policy remains focused on younger adults, but evidence that the majority of unplanned pregnancies occur among women aged 25 years and over,^1^ has prompted pleas for age-specific health promotion strategies.^29 30^

The independent associations of relationship type and duration with method use, and the suggestion that duration might be the stronger influence, has implications for policy and practice. Conventionally in clinical record-taking, data are collected on relationship type. Our findings suggest that there are benefits to be gained from asking about relationship duration in helping support women in their use of contraception, to help them consider their priorities and preferences at different life stages. Our findings highlight the complex nature of associations between age, partnership characteristics and method use, and signify that every woman needs to be assessed individually.

### Future research

Future research should recognise that the associations between relationship characteristics and contraceptive method use are more complex than might be suggested from aggregate data often used to assess the need for health promotional messages and public health interventions. Studies need to take account of both age and the nature of the relationships in which women use contraception.

10.1136/bmjsrh-2017-200037.supp1 Supplementary file 1

10.1136/bmjsrh-2017-200037.supp2 Supplementary file 2
