# Supplementary material for: Contraceptive method use among women and its association with age, relationship status and duration: findings from the third British National Survey of Sexual Attitudes and Lifestyles (Natsal-3)
Source: BMJ Sex Reprod Health. 2018 May 25;44(3):165–74. doi: 10.1136/bmjsrh-2017-200037 (PMC6225475; doi:10.1136/bmjsrh-2017-200037)
Supplement: Supplementary file 3 [file bmjsrh-2017-200037supp003.pdf]

Supplementary file 3: Distribution of partnership characteristics, by age group

	16-24		25-34		35-49		Total		
	n	% (95% CI)	n	% (95% CI)	n	% (95% CI)	Unweighted n	Weighted n	Weighted column %
**Relationship duration**									
1 day	272	18.8 (16.6,21.3)	204	10.3 (9.0,11.9)	91	7.2 (5.7,8.9)	567	353	11.2 (10.2,12.4)
>1 day <6 months	233	15.3 (13.4,17.5)	165	7.7 (6.5,9.2)	52	3.2 (2.4,4.3)	450	246	7.8 (7.1,8.7)
≥6 months <1 year	212	14.3 (12.4,16.3)	121	6.2 (5.0,7.6)	58	3.4 (2.6,4.6)	391	225	7.2 (6.4,8.0)
≥1 year <3 years	367	24.8 (22.5,27.3)	206	11.1 (9.5,12.9)	77	5.1 (4.1,6.5)	650	384	12.2 (11.2,13.3)
≥3years <5 years	224	15.4 (13.4,17.6)	253	13.3 (11.7,15.0)	79	6.6 (5.2,8.3)	556	347	11.0 (10.0,12.1)
≥5 years	167	11.4 (9.6,13.3)	914	51.4 (48.6,54.1)	761	74.5 (71.8,77.0)	1842	1588	50.5 (48.8,52.2)
**Relationship status**									
Recently met	70	5.1 (4.0,6.5)	82	4.1 (3.2,5.2)	29	1.9 (1.3,2.9)	181	109	3.5 (3.0,4.1)
Not steady	332	21.9 (19.5,24.5)	271	12.6 (10.9,14.5)	148	9.5 (8.0,11.3)	751	432	13.8 (12.6,14.9)
Steady, non-cohabiting	748	48.4 (45.5,51.3)	439	19.3 (17.5,21.3)	198	13.0 (11.2,15.2)	1385	765	24.3 (23.0,25.7)
Married/cohabiting	325	24.7 (22.3,27.2)	1071	64.0 (61.6,66.3)	743	75.5 (72.9,77.9)	2139	1838	58.5 (56.8,60.1)
**Total**									
Unweighted	1475	33.2	1863	38.6	1118	28.2	4456		100
Weighted	823	26.2 (24.8,27.6)	1014	32.3 (30.8,33.8)	1307	41.6 (39.8,43.4)		3143	100
