# Supplementary material for: Contraceptive method use among women and its association with age, relationship status and duration: findings from the third British National Survey of Sexual Attitudes and Lifestyles (Natsal-3)
Source: BMJ Sex Reprod Health. 2018 May 25;44(3):165–74. doi: 10.1136/bmjsrh-2017-200037 (PMC6225475; doi:10.1136/bmjsrh-2017-200037)
Supplement: Supplementary file 4 [file bmjsrh-2017-200037supp004.pdf]

Supplementary file 4 - Crude odds of using each contraceptive method group by partnership characteristics, stratified by age

	Unreliable or no method OR (95% CI)	Barrier methods OR (95% CI)	Oral and injectable hormonal methods OR (95% CI)	LARC OR (95% CI)
**16-24**				
**Relationship duration**				
1 day (ref.)	1	1	1	1
>1 day <6 months	0.28 (0.14,0.57)	1.28 (0.83,1.97)	1.33 (0.88,2.02)	0.84 (0.44,1.60)
≥6 months <1 year	0.61 (0.32,1.16)	0.46 (0.28,0.76)	2.18 (1.45,3.30)	1.11 (0.59,2.10)
≥1 year <3 years	0.35 (0.20,0.63)	0.39 (0.26,0.59)	2.33 (1.62,3.36)	1.67 (1.00,2.79)
≥3years <5 years	0.52 (0.28,0.97)	0.63 (0.39,1.01)	1.86 (1.22,2.84)	1.13 (0.61,2.10)
≥5 years	0.45 (0.21,0.97)	0.47 (0.28,0.80)	1.75 (1.13,2.70)	1.97 (1.06,3.69)
**Relationship status**				
Recently met (ref.)	1	1	1	1
Not steady	0.90 (0.34,2.33)	0.59 (0.33,1.06)	1.78 (0.98,3.23)	1.01 (0.43,2.37)
Steady, non-cohabiting	0.48 (0.20,1.19)	0.35 (0.20,0.62)	3.05 (1.73,5.35)	1.21 (0.54,2.69)
Married/cohabiting	0.85 (0.33,2.15)	0.31 (0.17,0.56)	2.43 (1.34,4.39)	1.59 (0.69,3.66)
**25-34**				
**Relationship duration**				
1 day (ref.)	1	1	1	1
>1 day <6 months	0.42 (0.24,0.73)	1.03 (0.55,1.91)	1.82 (1.10,3.03)	1.04 (0.54,2.03)
≥6 months <1 year	0.56 (0.28,1.14)	0.76 (0.38,1.51)	1.77 (1.05,2.99)	1.25 (0.63,2.47)
≥1 year <3 years	0.52 (0.30,0.87)	0.82 (0.48,1.41)	2.17 (1.36,3.45)	0.81 (0.41,1.59)
≥3years <5 years	0.60 (0.36,0.98)	0.56 (0.34,0.92)	2.43 (1.59,3.72)	0.89 (0.48,1.65)
≥5 years	0.48 (0.32,0.71)	0.77 (0.51,1.15)	1.87 (1.31,2.67)	1.33 (0.82,2.18)
**Relationship status**				
Recently met (ref.)	1	1	1	1
Not steady	1.40 (0.68,2.86)	0.71 (0.37,1.37)	.95 (0.55,1.66)	1.29 (0.56,2.96)
Steady, non-cohabiting	1.09 (0.54,2.21)	0.53 (0.28,1.01)	1.24 (0.73,2.09)	1.58 (0.72,3.49)
Married/cohabiting	1.06 (0.55,2.05)	0.72 (0.40,1.30)	1.02 (0.61,1.72)	1.57 (0.74,3.33)
**35-49**				
**Relationship duration**				
1 day (ref.)	1	1	1	1
>1 day <6 months	1.64 (0.77,3.51)	1.23 (0.48,3.19)	0.43 (0.16,1.14)	0.82 (0.30,2.27)
≥6 months <1 year	1.29 (0.63,2.63)	1.28 (0.49,3.31)	0.81 (0.35,1.86)	0.65 (0.25,1.71)
≥1 year <3 years	1.25 (0.63,2.47)	1.39 (0.58,3.35)	0.69 (0.30,1.62)	0.74 (0.30,1.86)
≥3years <5 years	1.95 (0.97,3.94)	0.94 (0.39,2.30)	0.68 (0.30,1.58)	0.50 (0.19,1.32)
≥5 years	0.93 (0.55,1.56)	1.38 (0.70,2.70)	0.94 (0.54,1.64)	0.88 (0.48,1.61)
**Relationship status**				
Recently met (ref.)	1	1	1	1
Not steady	5.39 (1.94,14.96)	0.61 (0.22,1.71)	1.08 (0.37,3.13)	0.22 (0.08,0.59)
Steady, non-cohabiting	3.65 (1.35,9.84)	0.82 (0.30,2.26)	1.35 (0.47,3.89)	0.26 (0.10,0.63)
Married/cohabiting	2.74 (1.03,7.23)	0.82 (0.32,2.11)	1.67 (0.63,4.42)	0.32 (0.14,0.73)
