# Supplementary material for: Contraceptive method use among women and its association with age, relationship status and duration: findings from the third British National Survey of Sexual Attitudes and Lifestyles (Natsal-3)
Source: BMJ Sex Reprod Health. 2018 May 25;44(3):165–74. doi: 10.1136/bmjsrh-2017-200037 (PMC6225475; doi:10.1136/bmjsrh-2017-200037)
Supplement: Supplementary file 5 [file bmjsrh-2017-200037supp005.pdf]

Supplementary file 5: Distribution of women reporting condom use by partnership characteristics, by age group

	16-24		25-34		35-49		Total		
	n	% (95% CI)	n	% (95% CI)	n	% (95% CI)	Unweighted	Weighted	
**Relationship duration**							n	n	% (95% CI)
1 day	87	33.6 (27.2,40.5)	52	27.8 (21.1,35.5)	16	17.2 (10.0,28.5)	156	97	27.5 (23.3,32.2)
>1 day <6 months	82	38.5 (31.6,45.8)	36	28.0 (19.0,39.2)	11	20.4 (11.3,33.9)	129	79	32.1 (27.2,37.4)
≥6 months <1 year	38	18.6 (13.3,25.4)	22	22.5 (13.8,34.5)	11	21.0 (11.9,34.5)	71	45	20.2 (15.7,25.6)
≥1 year <3 years	63	16.4 (12.7,21.0)	39	23.3 (16.7,31.4)	17	22.4 (13.6,34.7)	119	75	19.5 (16.3,23.1)
≥3years <5 years	51	24.0 (18.3,30.8)	46	17.6 (13.0,23.3)	16	16.4 (9.7,26.3)	113	68	19.6 (16.1,23.7)
≥5 years	29	18.6 (12.9,26.0)	197	22.8 (19.8,26.0)	157	21.9 (18.9,25.2)	383	349	22.0 (19.9,24.3)
**Relationship status**									
Recently met	29	44.8 (32.5,57.7)	20	30.4 (19.8,43.6)	7	25.4 (11.8,46.3)	56	38	34.7 (27.1,43.3)
Not steady	99	31.8 (26.5,37.7)	54	23.6 (17.3,31.3)	29	17.3 (11.9,24.4)	182	109	25.2 (21.8,29.0)
Steady, non-cohabiting	161	22.1 (18.8,25.9)	69	18.5 (14.4,23.3)	42	21.8 (15.7,29.5)	272	161	21.1 (18.6,23.9)
Married/cohabiting	61	19.5 (15.2,24.7)	249	23.8 (21.1,26.8)	150	21.4 (18.5,24.6)	460	405	22.1 (20.1,24.1)
**Total**									
Unweighted	350		392		228		970		
Weighted	204	28.6 (25.6,31.7)	234	32.7 (29.5,36.2)	276	38.7 (35.0,42.6)		714	
