# Supplementary material for: Contraceptive method use among women and its association with age, relationship status and duration: findings from the third British National Survey of Sexual Attitudes and Lifestyles (Natsal-3)
Source: BMJ Sex Reprod Health. 2018 May 25;44(3):165–74. doi: 10.1136/bmjsrh-2017-200037 (PMC6225475; doi:10.1136/bmjsrh-2017-200037)
Supplement: Supplementary file 6 [file bmjsrh-2017-200037supp006.pdf]

Supplementary file 6 - Adjusted odds ratios (with 95% confidence intervals) of using each category of contraceptive method according to relationship characteristics, stratified by age group

	Unreliable or no method OR (95% CI)	Barrier OR (95% CI)	Oral and injectable hormonal methods OR (95% CI)	LARCs OR (95% CI)
**16-24**				
**Relationship duration**				
1 day (ref.)	1	1	1	1
>1 day <6 months	0.31 (0.15,0.64)	1.56 (0.96,2.52)	1.11 (0.71,1.72)	0.83 (0.43,1.61)
≥6 months <1 year	0.65 (0.31,1.39)	0.60 (0.35,1.05)	1.74 (1.10,2.74)	1.08 (0.56,2.07)
≥1 year <3 years	0.35 (0.17,0.73)	0.52 (0.32,0.85)	1.89 (1.25,2.87)	1.59 (0.91,2.78)
≥3years <5 years	0.47 (0.21,1.05)	0.83 (0.48,1.46)	1.57 (0.98,2.52)	1.06 (0.54,2.08)
≥5 years	0.36 (0.14,0.94)	0.65 (0.34,1.23)	1.55 (0.92,2.60)	1.78 (0.88,3.61)
**Relationship status**				
Recently met (ref.)	1	1	1	1
Not steady	1.20 (0.45,3.16)	0.69 (0.37,1.26)	1.45 (0.78,2.69)	0.90 (0.38,2.13)
Steady, non-cohabiting	0.85 (0.31,2.34)	0.46 (0.24,0.89)	2.12 (1.14,3.96)	0.99 (0.43,2.26)
Married/cohabiting	1.62 (0.52,5.10)	0.47 (0.22,0.97)	1.59 (0.80,3.15)	1.11 (0.45,2.77)
**25-34**				
**Relationship duration**				
1 day (ref.)	1	1	1	1
>1 day <6 months	0.38 (0.20,0.72)	1.20 (0.63,2.30)	1.89 (1.11,3.22)	0.93 (0.48,1.83)
≥6 months <1 year	0.48 (0.23,1.02)	0.84 (0.41,1.69)	2.08 (1.19,3.62)	1.04 (0.49,2.19)
≥1 year <3 years	0.44 (0.24,0.79)	0.83 (0.47,1.47)	2.66 (1.57,4.50)	0.69 (0.34,1.42)
≥3years <5 years	0.50 (0.28,0.91)	0.51 (0.30,0.88)	3.22 (2.00,5.18)	0.77 (0.38,1.55)
≥5 years	0.40 (0.24,0.68)	0.66 (0.43,1.04)	2.57 (1.68,3.94)	1.18 (0.65,2.13)
**Relationship status**				
Recently met (ref.)	1	1	1	1
Not steady	2.06 (0.90,4.70)	0.83 (0.42,1.63)	0.61 (0.33,1.11)	1.30 (0.53,3.15)
Steady, non-cohabiting	1.91 (0.79,4.63)	0.66 (0.33,1.31)	0.65 (0.36,1.20)	1.67 (0.69,4.00)
Married/cohabiting	1.99 (0.84,4.72)	1.11 (0.57,2.17)	0.48 (0.25,0.90)	1.46 (0.60,3.53)
**35-49**				
**Relationship duration**				
1 day (ref.)	1	1	1	1
>1 day <6 months	1.74 (0.80,3.77)	1.20 (0.45,3.22)	0.41 (0.15,1.12)	0.83 (0.30,2.31)
≥6 months <1 year	1.27 (0.60,2.70)	1.28 (0.43,3.78)	0.70 (0.28,1.72)	0.80 (0.28,2.26)
≥1 year <3 years	1.24 (0.61,2.54)	1.43 (0.54,3.80)	0.56 (0.23,1.39)	0.93 (0.34,2.53)
≥3years <5 years	2.05 (0.97,4.33)	0.98 (0.37,2.59)	0.52 (0.22,1.25)	0.61 (0.20,1.84)
≥5 years	1.06 (0.58,1.93)	1.45 (0.66,3.17)	0.66 (0.33,1.31)	0.99 (0.44,2.24)
**Relationship status**				
Recently met (ref.)	1	1	1	1
Not steady	5.38 (1.92,15.07)	0.55 (0.19,1.59)	1.21 (0.38,3.82)	0.23 (0.08,0.64)
Steady, non-cohabiting	3.34 (1.17,9.51)	0.69 (0.21,2.23)	1.75 (0.52,5.86)	0.28 (0.10,0.80)
Married/cohabiting	2.96 (1.03,8.50)	0.63 (0.21,1.90)	2.05 (0.63,6.63)	0.31 (0.11,0.90)
