# Supplementary figures and images for: Contraceptive method use among women and its association with age, relationship status and duration: findings from the third British National Survey of Sexual Attitudes and Lifestyles (Natsal-3)
Source: BMJ Sex Reprod Health. 2018 May 25;44(3):165–74. doi: 10.1136/bmjsrh-2017-200037 (PMC6225475; doi:10.1136/bmjsrh-2017-200037)

## Supplementary file 1: Study sample

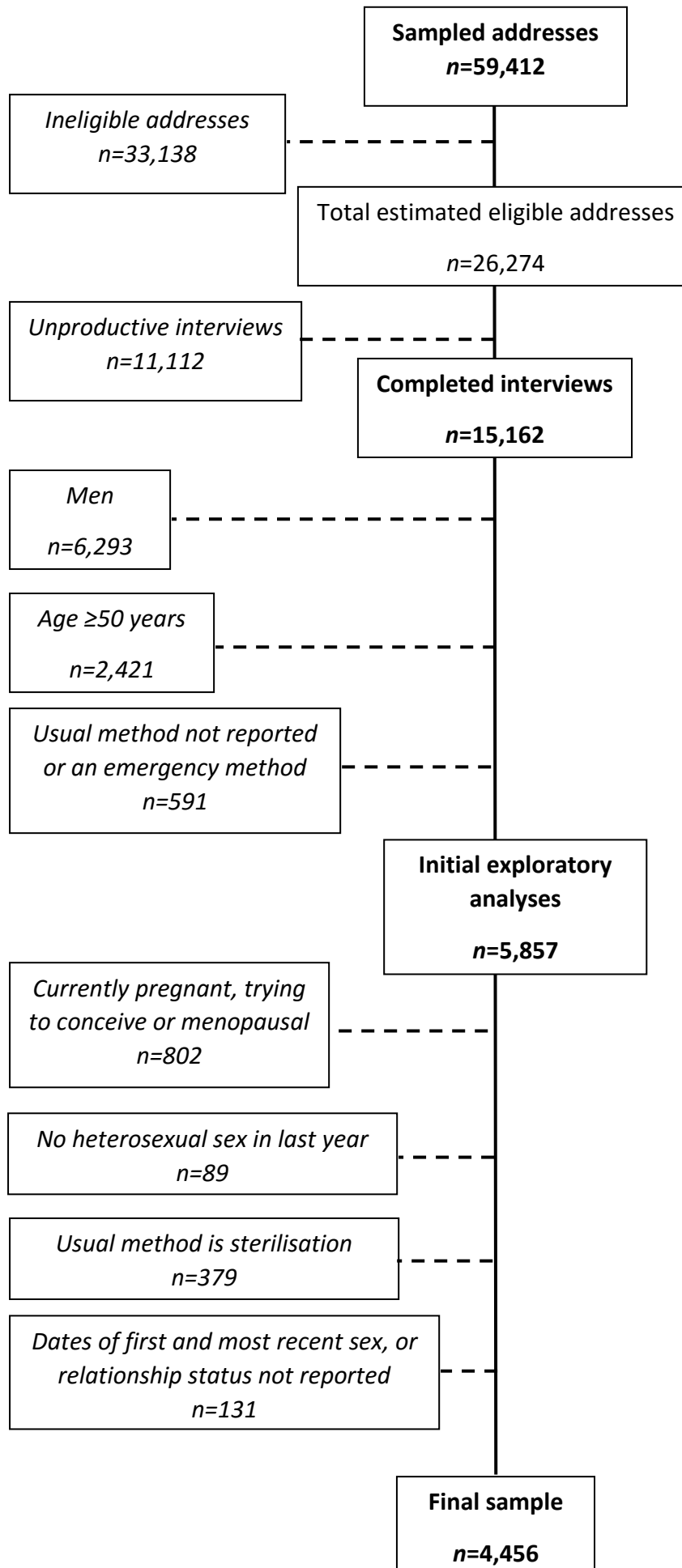

Supplement: Supplementary file 1 [file bmjsrh-2017-200037supp001.pdf]
