# Supplementary material for: Contraceptive method use among women and its association with age, relationship status and duration: findings from the third British National Survey of Sexual Attitudes and Lifestyles (Natsal-3)
Source: BMJ Sex Reprod Health. 2018 May 25;44(3):165–74. doi: 10.1136/bmjsrh-2017-200037 (PMC6225475; doi:10.1136/bmjsrh-2017-200037)
Supplement: Supplementary file 2 [file bmjsrh-2017-200037supp002.pdf]

Supplementary file 2: Distribution of participants according to demographic and partnership characteristics, and type of method of contraception used usually (with 95% confidence intervals), for those meeting the study inclusion criteria and those not

Variable		Sample meeting all inclusion criteria % (95% CI)	Sample not meeting inclusion criteria % (95% CI)
**Gender**			
	Male	0.0	62.5 (61.4,63.5)
	Female	100.0	37.5 (36.5,38.6)
**Age**			
	16-24	26.2 (24.8,27.6)	13.5 (12.9,14.1)
	25-34	32.3 (30.8,33.8)	14.5 (13.8,15.2)
	35-49	41.6 (39.8,43.4)	25.8 (24.8,26.9)
	>50	0	46.2 (45.0,47.3)
**Usual method of contraception**			
	Unreliable or no method	24.3 (22.7,26.0)	45.0 (43.8,46.1)
	Barrier	22.9 (21.5,24.4)	14.2 (13.5,15.1)
	Oral and injectable hormonal	36.6 (35.0,38.3)	11.1 (10.4,11.8)
	LARC	16.1 (14.8,17.5)	4.5 (4.0,5.0)
	Missing	0	25.2 (24.3,26.2)
**Relationship duration**			
	1 day	11.2 (10.2, 12.4)	7.9 (7.4, 8.5)
	>1 day <6 months	7.8 (7.1,8.7)	4.1 (3.8,4.5)
	≥6 months <1 year	7.2 (6.4,8.0)	3.6 (3.2,4.0)
	≥1 year <3 years	12.2 (11.2,13.3)	6.1 (5.7,6.6)
	≥3years <5 years	11.0 (10.0,12.1)	6.1 (5.6,6.6)
	≥5 years	50.5 (48.8,52.2)	59.5 (58.4,60.7)
	Missing	0	12.5 (11.8,13.3)
**Relationship status**			
	Recently met	3.5 (3.0,4.1)	3.6 (3.3, 4.0)
	Not steady	13.8 (12.6,14.9)	7.9 (7.4, 8.5)
	Steady, non-cohabiting	24.3 (23.0,25.7)	13.3 (12.6,14.0)
	Married/cohabiting	58.5 (56.8,60.1)	65.1 (64.0, 66.2)
	Missing	0	10.0 (9.4,10.8)
**Total (weighted)**		3144	12018
**Total (unweighted)**		4456	10706
